# Supplementary material for: Dietary manipulation of the gut microbiome in inflammatory bowel disease patients: Pilot study
Source: Gut Microbes. 2022 Mar 20;14(1):2046244. doi: 10.1080/19490976.2022.2046244 (PMC8942410; doi:10.1080/19490976.2022.2046244)
Supplement: Supplemental Material [file KGMI_A_2046244_SM7064.zip › 20211209_Supplementary Figures Legends.docx]

**Supplementary Figure 1.** A) Shannon diversity and B) T-distributed stochastic neighbor embedding analysis shows that both CD and UC participants showed a personalized microbiome that clustered individually rather than by disease phenotype.

**Supplementary Figure 2**. A) Shannon diversity and B) tSNE clustering for samples at Baseline (BSL) and during Intervention (INT)

**Supplementary Figure 3**. Levels of circulatory inflammatory markers at baseline (BSL; N=9, red circles) and at the end of the intervention (INT; N=9, blue circles).
