# Supplementary figures and images for: Dietary manipulation of the gut microbiome in inflammatory bowel disease patients: Pilot study
Source: Gut Microbes. 2022 Mar 20;14(1):2046244. doi: 10.1080/19490976.2022.2046244 (PMC8942410; doi:10.1080/19490976.2022.2046244)

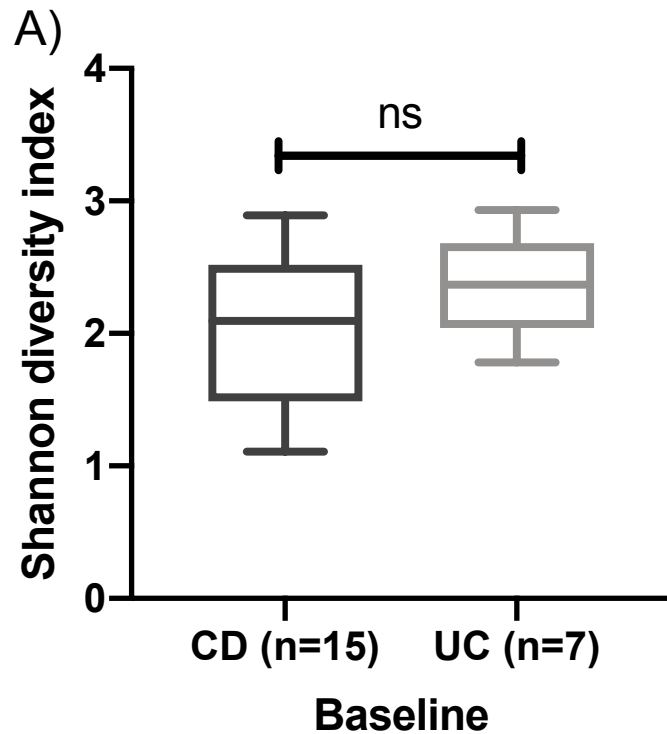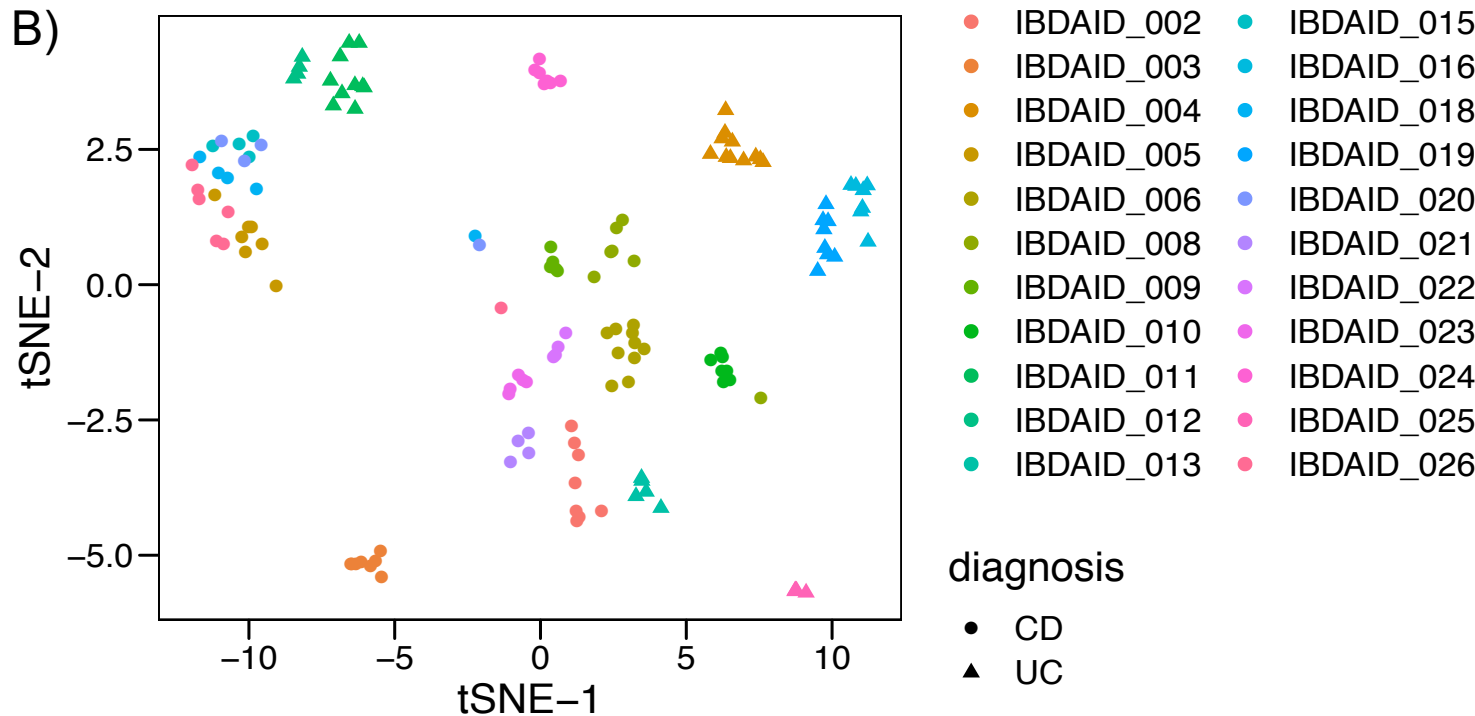

Supplement: Supplemental Material [file KGMI_A_2046244_SM7064.zip › suppl_fig 1_new.pdf.pdf]

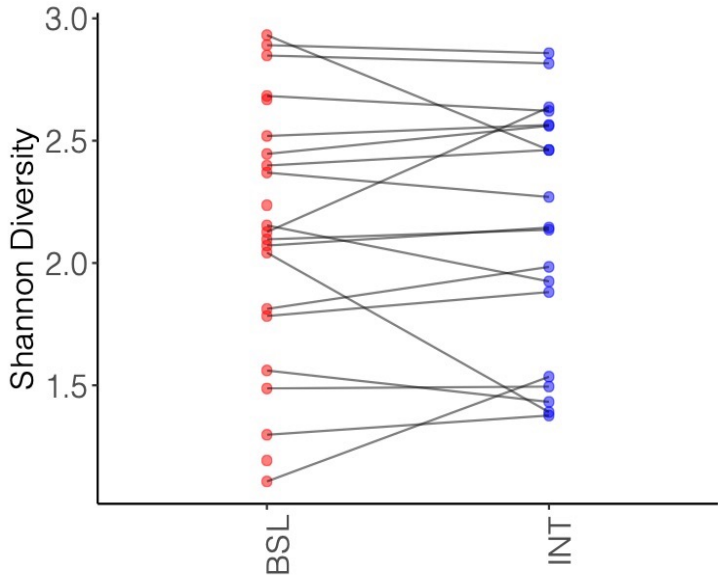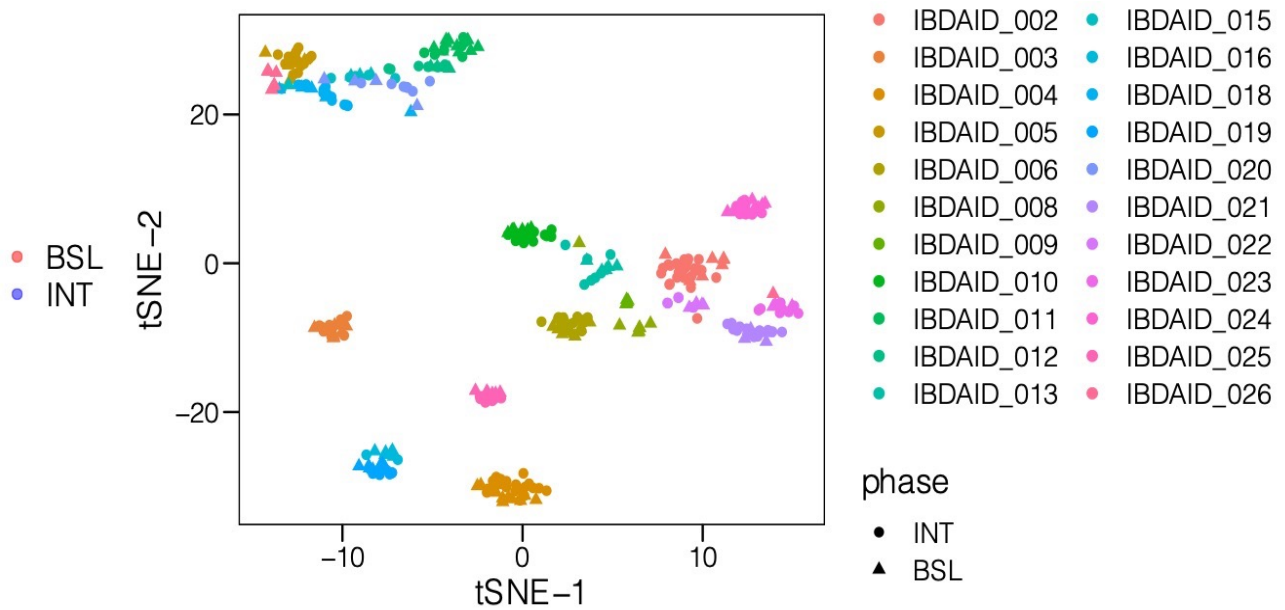

Supplement: Supplemental Material [file KGMI_A_2046244_SM7064.zip › suppl_fig 2.pdf.pdf]

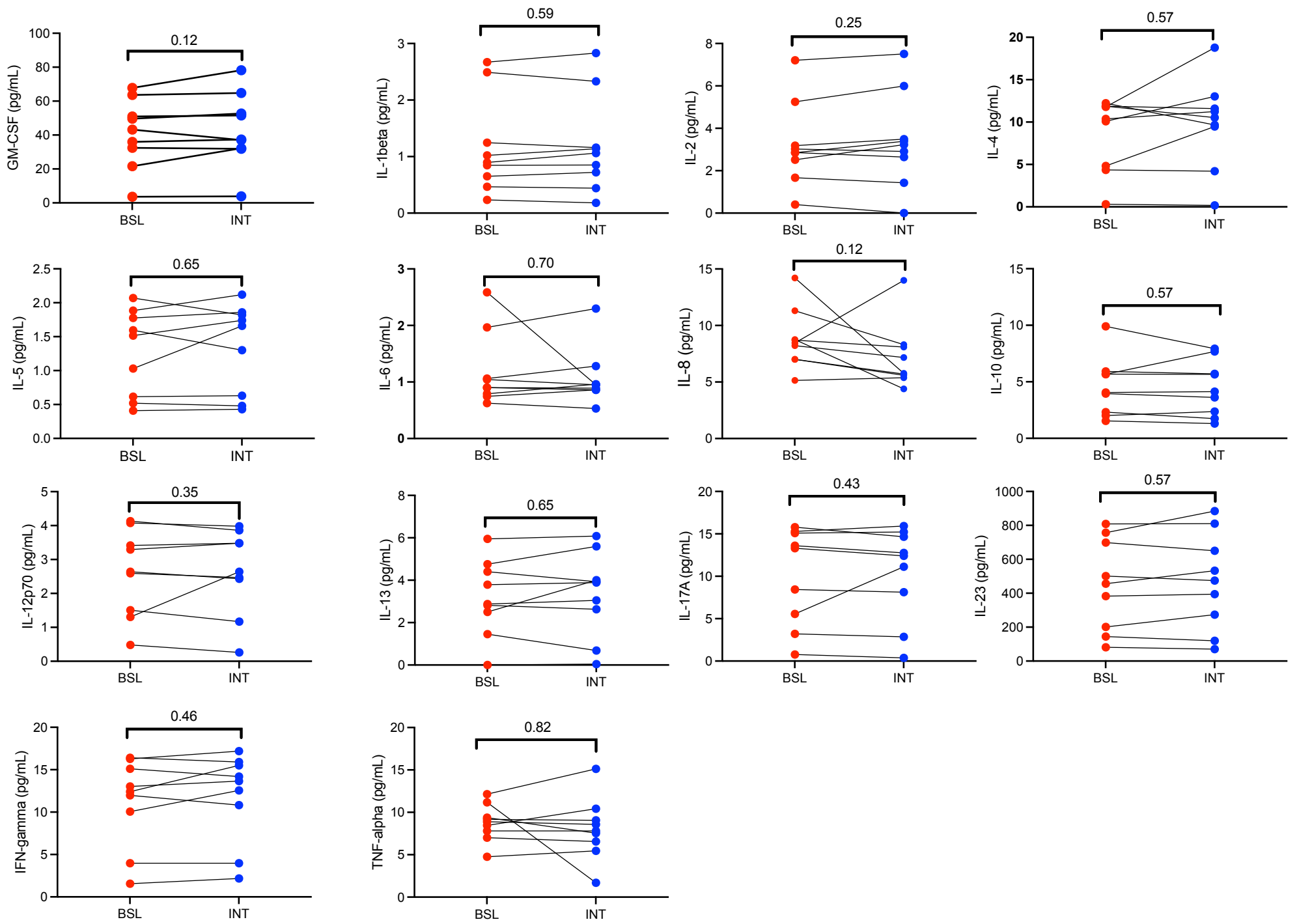

Supplement: Supplemental Material [file KGMI_A_2046244_SM7064.zip › suppl_fig 3_new.pdf.pdf]
